# Tsunami evacuation simulation using geographic information systems for homecare recipients depending on electric devices

**DOI:** 10.1371/journal.pone.0199252

**Published:** 2018-06-21

**Authors:** Hisao Nakai, Tomoya Itatani, Ryo Horiike, Kaoru Kyota, Keiko Tsukasaki

**Affiliations:** 1 Nursing Department, Kanazawa Medical University, Kanazawa, Ishikawa Prefecture, Japan; 2 Division of Health Sciences, Doctoral Course of Graduate School of Medical Pharmaceutical and Health Sciences, Kanazawa University, Kanazawa, Ishikawa Prefecture, Japan; 3 Medical Policy Section, Health Policy Department, Kochi, Kochi Prefecture, Japan; University of Memphis, UNITED STATES

## Abstract

Tsunamis cause direct damage to property and destroy infrastructure. In addition, power outages can lead to death, especially for patients who rely on medical equipment requiring a power supply. Recently, Nankai Trough Earthquakes have been predicted, and much effort has been put into developing countermeasures in Japan. Kochi City on Shikoku Island is expected to suffer in the event of a large tsunami. The present study identifies individuals living in Kochi who need evacuation assistance and depend on electrical medical devices, simulates evacuation behavior and inundation during a tsunami using a geographic information system (GIS), and considers the usefulness of such a GIS. We asked caregivers, including visiting nurses, to introduce us to homecare recipients who rely on a ventilator, an endotracheal suction device, or other medical devices requiring electric power. We received introductions to 52 homecare recipients. Using a GIS, we plotted the area of predicted inundation and the locations of homecare recipients, nursing stations, and welfare evacuation shelters. We predicted evacuation routes, and then analyzed the time difference between the time required for evacuation and tsunami arrival at a welfare evacuation shelter. To measure the effects of the main parameters, we conducted both one-way and multi-way sensitivity analysis. In the event of a tsunami, eight of the homecare recipients living in the forecasted inundation areas in Kochi may face delayed evacuation. Among homecare recipients facing a high possibility of escape delay, 95.2% lived more than 1,800 m from the nearest welfare evacuation shelter. We found that individual evacuation behavior can be simulated by specifying the residence of a homecare recipient and the evacuation route using a GIS.

## Introduction

Among the possible disasters, tsunamis have recently caused the highest number of casualties [1]. In 2004, a tsunami killed approximately 226,000 people in 12 countries [2]. The young and elderly are especially vulnerable groups during disasters [3][4]. Older adults are potentially vulnerable due to pre-existing medical issues, with increased mortality rates during and after disasters [5][6]. Also, individuals with special medical needs tend to have increased mortality rates during disasters, so consideration of availability of transport, shelter, and caregivers is necessary during emergency planning [7]. Disasters not only cause direct damage, such as crushing due to building collapse, but they also destroy infrastructure, which affects residents. Patients with chronic disease are particularly susceptible to infrastructure destruction. If infusion or medical treatment is interrupted by a power outage, for example, patients with chronic diseases are severely affected and they have an increased risk of death due to serious complications [8]. Power outages can directly lead to death, especially for patients who need medical equipment requiring a power supply.

In Japan, two major earthquakes, namely the 2011 Great East Japan Earthquake and 2016 Kumamoto Earthquake, occurred within only 5 years of each other and caused serious damage. Approximately 20,000 people died in the 2011 earthquake and tsunami disaster [9]. In the earthquake, users of artificial ventilators and oxygen concentrators living in districts that were not affected by the tsunami died as a result of power outages [10]. In recent years, earthquakes with seismic epicenters from Shizuoka Prefecture to Shikoku and Kyushu islands, the so-called Nankai Trough Earthquakes, have been predicted. Because these earthquakes are expected to be capable of creating large tsunamis, much effort has been put into developing countermeasures [11]. Several cities, including Kochi, are expected to suffer the effects of a large tsunami when a Nankai Trough Earthquake occurs. Kochi is a city with a population of approximately 330,000 located on the Pacific side of Shikoku Island. Tsunamis reached Kochi following the Showa Nankai Earthquake in 1946 and an earthquake that struck South America in 2010 [12][13]. In Kochi, the probability of an earthquake associated with the Nankai Trough occurring within the next 30 years is estimated to be around 70% [14]. If the currently assumed biggest earthquake strikes, it is estimated that a tsunami will reach Kochi within 20 minutes [15], and there will be long-lasting flooding due to subsidence of the ground even after the tsunami recedes [16].

To safeguard individuals who need electric power to live or need support for their chronic illness, the Japanese government requests that municipalities prepare a list of residents requiring support and support these individuals during disasters [17]. In response to this, municipalities call on residents to determine whether they need assistance in the event of a disaster, aggregate the replies requesting assistance and the existing materials stored by municipalities, and prepare a list of homecare recipients who need support in an evacuation. For those who need special consideration, including regular users of medical equipment, municipalities implement countermeasures, including calling for the stockpiling of evacuation goods, selection of caregivers, and preparation of individual evacuation plans. In areas where tsunami damage is expected, measures should be taken not only for healthy people but also for those who need special assistance, such as those suffering chronic diseases, disabled people, pregnant women, the elderly, and children. It is especially urgent to take measures for users of medical equipment requiring electric power. After the Great East Japan Earthquake, the use of external battery bags for homecare recipients came to be adopted as standard nursing care [18], and battery-powered ventilators had to be supplied by medical device manufacturers [19]; however, ventilator battery life was about 6 hours maximum, which is not enough time for a rescue team to reach the patient.

To provide a concrete countermeasure against a large tsunami disaster, a geographic information system (GIS) is useful for comprehensively displaying the status of residents, including their mobility and location on a map, and in planning individual countermeasures according to each resident’s status.

GISs have been used since the early 1990s, and their use is spreading in the field of public health [20]; for example, studies have used GIS to estimate the risk of spreading infection [21] and to combat diabetes by visualizing the diabetes morbidity rate [22]. Regarding research on use of GIS in disasters, Stefan et al. studied the health needs in areas most affected by flood damage due to typhoon Allison and demonstrated the usefulness of GIS utilization [23]. Additionally, Jacqueline et al. estimated the vulnerable population by visualizing the expected landslide and liquefaction zone during an earthquake [24]. In the United States, a system that combines behavioral risk factor surveillance and a GIS has been considered in evaluating the need for quick response in the event of a natural disaster [8]. Meanwhile, no study has used a GIS to examine the evacuation behavior of homecare recipients using life-support devices that require an external power supply. To examine the evacuation behavior in the event of a disaster, it is necessary to consider the travel speed and evacuation route of such homecare recipients. By conducting a GIS simulation, a detailed and more effective evacuation behavior study can be conducted.

In Kochi, disaster countermeasures are urgently needed for homecare recipients who need an electric power supply and live in areas where tsunamis are expected. This study identified those vulnerable groups in Kochi, simulated evacuation behavior and vulnerabilities during a tsunami using a GIS, and considered the usefulness of a GIS for disaster response.

## Method

### Data collection

From May 2015 to February 2016, we collected data on homecare recipients in Kochi who require electric power to stay alive. We asked health care professionals, including nurses, public health nurses, and care managers who are in charge of daily homecare recipients, to introduce us to homecare recipients who met specific conditions. The conditions were that the homecare recipient uses (1) a ventilator (invasive or noninvasive), (2) an endotracheal suction device, or (3) other medical equipment requiring an external power source. The frequency of use of medical devices was generally set to at least once a day. We then received introductions to 52 homecare recipients from health care professionals and collected data from them. We asked the health care professionals for an interview to complete a K-DiPS Checklist. This checklist allows homecare recipients and their families to grasp equipment, materials, and important points necessary during a disaster by filling in a form with health care professionals who are in charge of their daily living support [25].

### Visualization using a GIS

In a GIS, each type of information on a map can be gathered into a single layer. It is a characteristic of a GIS that several forms of information are layered onto a single map, and we can centrally view all the information by overlapping the layers.

**Fig 1:**
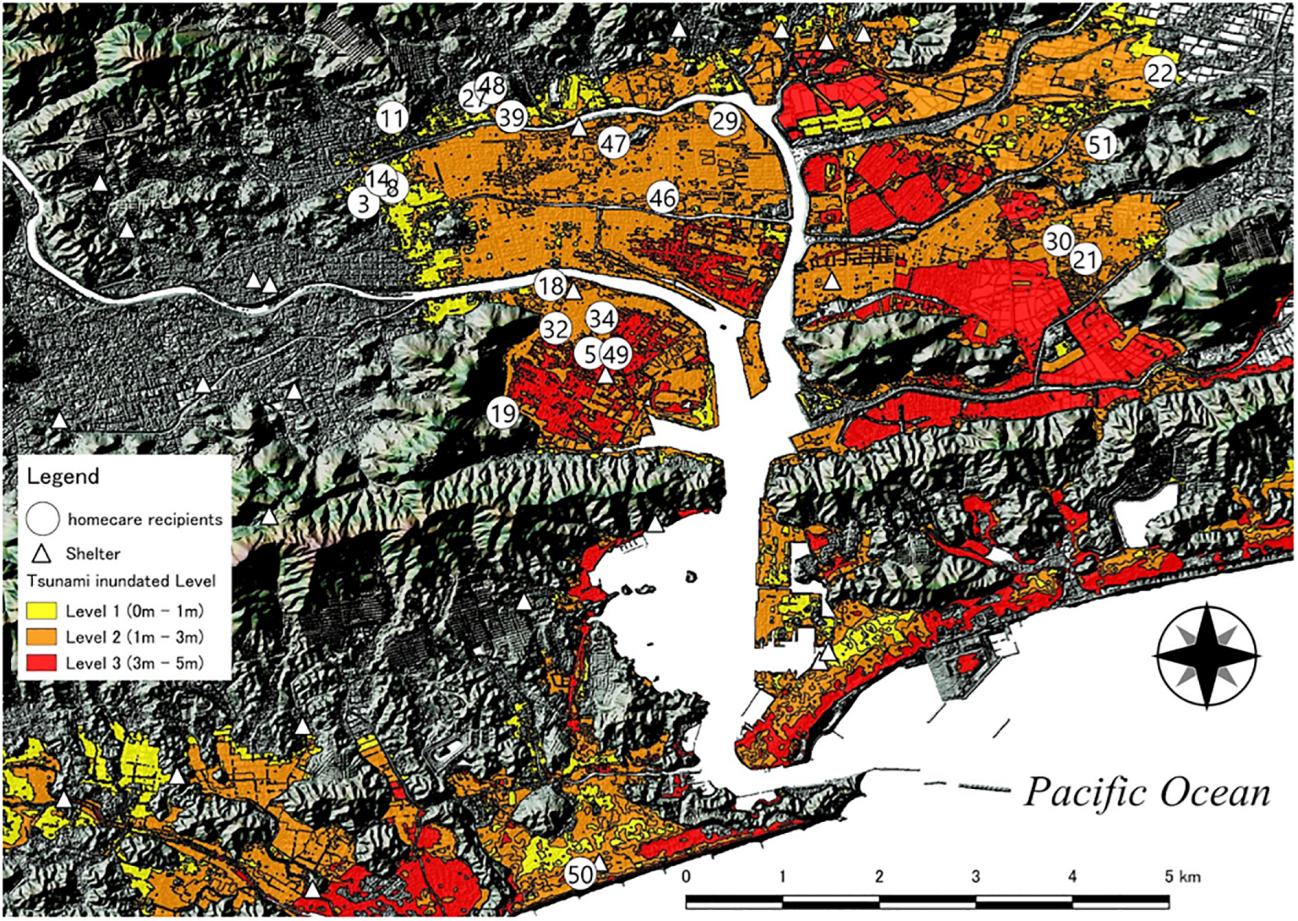
Area of predicted tsunami inundation and locations of homecare recipients. Fig 1 depicts areas at risk for flooding by a tsunami in Kochi. Fig 1 was drawn in the GIS by superimposing the layer describing the area of predicted flooding and the layer locating homecare recipients and welfare evacuation shelters. The sea is on the south side of Fig 1 (downward), and a tsunami will proceed from south to north (upward).

**Fig 2:**
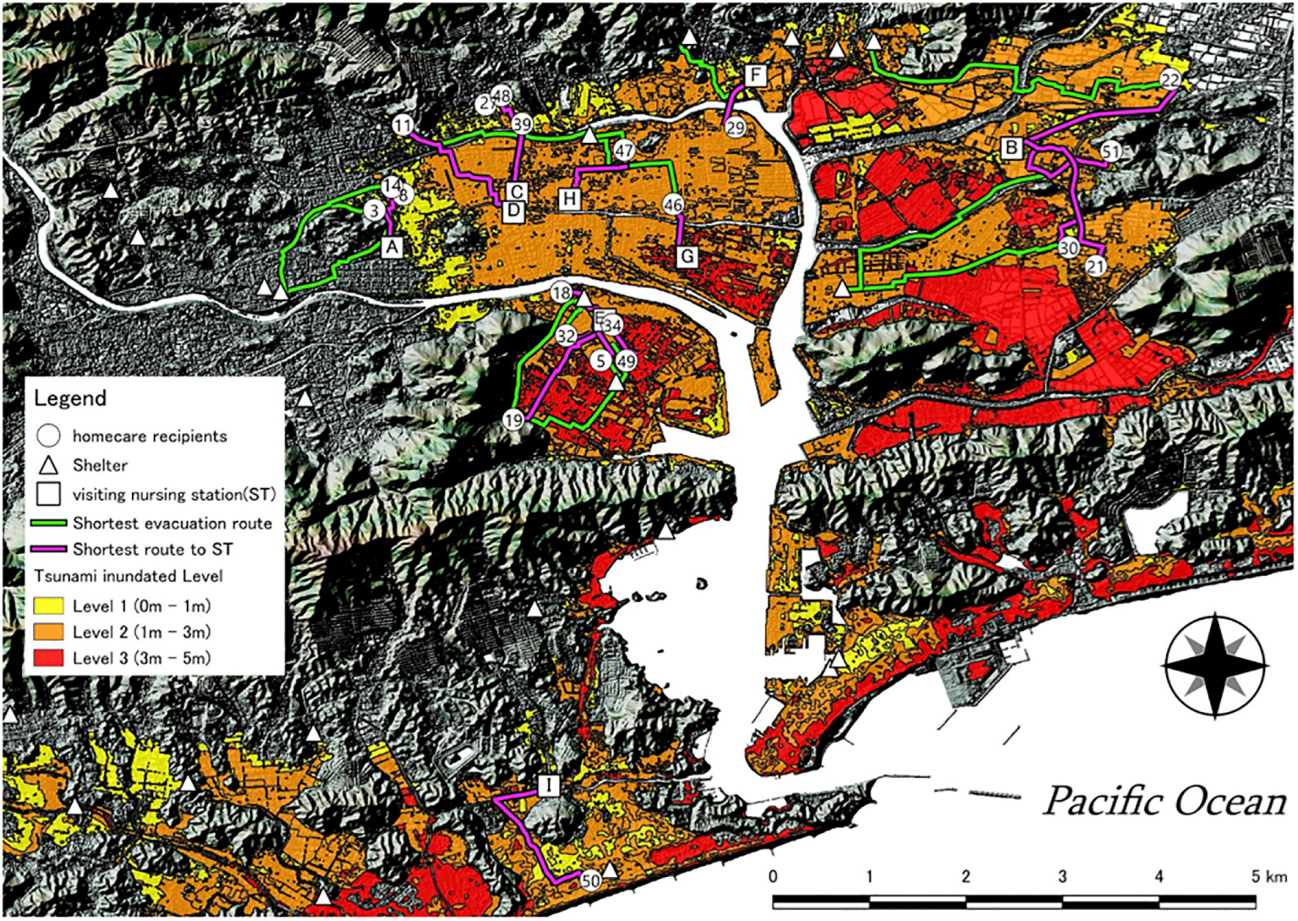
Routes from the homes of homecare recipients to welfare evacuation shelters and routes of visiting nurses traveling to homecare recipients. Pink lines in Fig 2 represent routes connecting each homecare recipient and the nearest visiting nursing station according to the shortest distance. Evacuation routes connecting welfare evacuation shelters and the homes of homecare recipients are depicted in green on the map.

We first obtained the addresses of nursing stations and welfare evacuation shelters in Kochi [26][27]. We assumed that visiting nurses would support the rescue of homecare recipients in the event of a disaster. The addresses of homecare recipients, nursing stations, and welfare evacuation shelters were then converted to location information using a geocoding service. As a privacy consideration, the positions of the homecare recipients were kept only on a town-level plot. We next obtained a base map for an elevation of 5 m using a numerical elevation model for the survey area, and created a shadow map layer based on this mesh. We then created a layer plotting the area of predicted inundation for the residential areas of homecare recipients. By overlaying these three layers (Fig 1), we visually checked for homecare recipients who need evacuation and examined evacuation routes (Fig 2).

Geographical analysis was conducted using QGIS version 2.18.3. For evacuation route analysis, we used the QGIS Road Graph Plugin. Road information that was the basis of evacuation routes was downloaded as polylines from Open Street Map. QGIS is open-source software that can be used to edit and analyze geographic information.

### Parameter setting

#### Time required for the visiting nurse to travel from a nursing station to homecare recipients

Potential rescuers, that is, individuals who might aid homecare recipients during a disaster, include members of the fire brigade, neighborhood residents, visiting nurses, nursing care helpers, and care managers. In emergencies generally, members of the fire brigade transport people using ambulances with life support equipment. However, during a disaster in which many people require rescue simultaneously, rescue by the fire brigade would not be expected. The government has issued guidelines for “supporting neighboring residents and related organizations” to provide support to those who need it when evacuating during a disaster; however, who is supposed to provide support depends on the judgment of people at the site. In addition, because the homecare recipients included in this survey use medical equipment requiring a power supply, it is difficult for rescuers to support evacuation while operating the medical equipment, unless they are a medical professional. It is therefore unreasonable to expect evacuation support from neighboring residents at present, and for the same reason, evacuation support from care helpers and care managers would not be expected. In the present study, therefore, we assumed that visiting nurses travel from visiting nursing stations to the homes of homecare recipients during the disaster and effect their evacuation. Certainly, in disasters, disabled people, elderly, and children have high priority for rescue. Among them, we believe that patients who are using ventilators should be given the highest priority. Elderly people can also be rescued by people other than medical staff, and children can be carried to safety by any adult. However, if a visiting nurse is going to rescue a homecare recipient, it is necessary to prepare a plan to take such action beforehand. Additionally, we assumed that during a disaster a visiting nurse travels from the nearest visiting nursing station to provide support. Although the visiting nursing station in charge of the homecare recipients may not be the nearest station, the nearest station is in charge in most cases and nearby stations are in charge in other cases. Actually, no survey has investigated the intention of nurses as to whether they would provide rescue support during a disaster. In this study, based on the behavior of nurses at the time of the Great East Japan Earthquake [28], we assumed nurses as rescuers. Otherwise, it is extremely difficult to rescue a homecare recipient in the current situation.

The distance from the station to the homecare recipient was calculated as follows. First, we obtained position information for visiting nursing stations and visually confirmed the positional relation between the visiting nursing station and the homecare recipients using the GIS. Next, the shortest route distance from the nearest station to homecare recipients was calculated according to the Open Street Map polyline and Road Graph Plugin.

We interviewed stations and found that visiting nurses use cars in most cases. Although some nurses use bicycles in urban areas, their traveling speed is almost the same as that of cars, and we therefore assumed that visiting nurses travel by car in this study. According to a survey, the travel speed was set to 360 m per minute, which is the average travel speed in Kochi [29]. Using this speed and the distance from the visiting nursing station to homecare recipients determined by the GIS, we calculated the time for the visiting nurse to reach the homecare recipient.

#### Time required for transfer

The national association for home-visiting nursing care conducted a survey on the time required for visiting nurses to assist patient transfer [30]. According to that survey, the average time taken for a visiting nurse to independently assist in transfer was 4.9 minutes. In the survey, 17.1% of homecare recipients used ventilators. In our survey, because everyone needing support uses medical equipment, such as a ventilator or suction device, it may take longer than the result of the previous study to complete the transfer. However, the result of that study is the most suitable value available for our survey, and our study therefore used the same transfer time.

#### Time required to travel from a homecare recipient’s residence to a shelter

In terms of the means of transporting a homecare recipient from their home to an evacuation shelter, we supposed that rescuers (i.e., visiting nurses) would transport homecare recipients using wheelchairs. The ventilator currently in use at residences is very compact, weighing 5 kg or less, and it is portable by wheelchair. Also, we confirmed that all the ventilators used by the subjects in this study are portable. The government has designated shelters to which earthquake-resistant measures have been applied [31], providing a safe space even when flooded for people who use medical equipment needing a power supply. Accordingly, we assumed that the homecare recipient evacuates to the welfare evacuation shelter that is physically closest to their home. However, we also assumed that if the nearest welfare evacuation shelter is located in an area with a flood level of 3, the homecare recipient will not evacuate to that welfare evacuation shelter but to the shelter nearest their home located in an area with a flood level of 1 or 2, because during a tsunami it is dangerous to go to any location where the flood level is 3.

According to a previous study investigating the traveling speed just before the arrival of the tsunami associated with the Great East Japan Earthquake, the walking speed of someone accompanying a disabled person is 1.88 km/h on average [32]. This speed was calculated from the distance to a welfare evacuation shelter and the time taken by people having difficulty walking, such as physically handicapped or seriously ill people, to walk that distance. This situation fits our study, so we used 1.88 km/h for the speed of travel to a welfare evacuation shelter by the homecare recipient and visiting nurse. Using this speed and the distances from the homes of the homecare recipients to the evacuation shelters determined by the GIS, we calculated the times for the homecare recipients to reach the welfare evacuation shelters.

#### Arrival time of a 30-centimeter tsunami at shelters

As discussed in the next section, the main outcome of this study is the time difference between tsunami arrival and evacuation. Next, we predicted the time needed for a tsunami with a flow depth of 30 cm to reach an evacuation shelter. This was done by referring to National Land Numeral Information (Tsunami Inundation Assumption) from the Japanese government for an earthquake with the maximum seismic intensity of 9. These public data give the predicted time at which a 30-cm tsunami flow arrives in each area of Kochi. National Land Numeral Information was created to support the development and implementation of National Land Plans, such as the National Land Formation Plan and National Land Use Plan. It is also expected to be widely used by local governments and research institutes, and it is provided free of charge on the internet [15][33].

The main outcome of this study is the time difference between the time required for evacuation and tsunami arrival at an evacuation shelter, and the number of homecare recipients having a negative value for this difference. The time required for evacuation is the total time, when a disaster occurs, for a visiting nurse rescuer to travel from the visiting nursing station to the residence of the homecare recipient, transfer the recipient to a wheelchair, and push the wheelchair from the homecare recipient’s home to an evacuation shelter. The situation that the tsunami reaches the welfare evacuation shelter before evacuation is complete is such that the welfare evacuation shelter will be flooded before the evacuation and the homecare recipients cannot complete their evacuation. We refer to this state as the delayed escape, which means that homecare recipients are in lethal situations in which they are dragged away by the tsunami or their life support equipment stops due to power outage or inundation.

### Sensitivity analysis

To measure the effects of the main parameters, we conducted both one-way and multi-way sensitivity analyses. The parameters used in the sensitivity analyses were (1) the speed at which the visiting nurse travels to a homecare recipient, (2) the time required for the visiting nurse to move the homecare recipient from their bed to a wheelchair, (3) the speed at which the rescuer and homecare recipients travel to the welfare evacuation shelter, and (4) the time it takes for the tsunami to arrive at the welfare evacuation shelter. The parameters were varied from 0.5 times to 2.0 times their base values to analyze the change in the time difference between the homecare recipient reaching a welfare evacuation shelter and the time the tsunami reaches the shelter. In the multi-way sensitivity analysis, the time differences of combinations of all four main parameters were confirmed using the lower and upper limits of each parameter.

### Ethical considerations

In gathering the data for this study, we explained the purpose of the survey to the homecare recipients, families, and health care professionals who are in charge of daily care by oral explanations and written documents. We also informed participants that participation in the survey was voluntary, there was no disadvantage in refusing to cooperate with the survey, and participants could stop cooperating at any time during the investigation. We received signed informed consent from homecare recipients and health care professionals. This study was carried out with the approval of the Kanazawa University Medical Ethics Association (No. 594).

In addition, during analysis, the locations of the homes of homecare recipients were depicted at the town level so as not to be able to identify the locations of individuals. We plotted the exact locations of visiting nursing stations and welfare evacuation shelters because they are publicly available.

## Results

### Predicted inundation area and locations of homecare recipients, shelters, and nursing stations

There were 52 homecare recipients in this study, of which 21 (40.4%) reside in the area of predicted tsunami inundation. The breakdown was 7 inhabitants (33.3%) for inundation level 1, 11 (52.3%) for inundation level 2, and 4 (19.0%) for inundation level 3. There were 33 welfare evacuation shelters in Kochi that could accommodate those who require life support, of which 17 (51.5%) were located in the forecast area of tsunami inundation. Three of those shelters were located inundation level 3 areas (17.6%). There were nine home visiting nursing stations near homecare recipients, and six (66.7%) were located in the area of predicted tsunami inundation.

### Time differences between evacuation and tsunami arrival (the main outcome) and locations of homecare recipients

Eight (38.1%) of the 21 homecare recipients who lived in the tsunami inundation forecast area were estimated to have a negative difference between the time required for evacuation and the predicted arrival time of the tsunami, that is, a delayed escape. The magnitude of the escape delay ranged from −4.3 to −104.8 minutes (Table 1).

**Table 1:** Difference between arrival times of homecare recipients and the tsunami at shelters.

Parameter		Seismic intensity distribution	Maximum inundation level	Distance from the nursing station to the homecare recipient	Time required for the visiting nurse to arrive at the homecare recipient’s home (at a speed of 360 m/min)	Time required to move the homecare recipient from their bed to a wheelchair	Distance from an individual homecare recipient’s home to a welfare evacuation shelter	Time required to travel from a homecare recipient’s home to an evacuation shelter (at a speed of 33.3 m/min)	Total time required to travel to an evacuation shelter	Time for a 30-cm tsunami to reach a welfare evacuation shelter	Time difference between arriving at an evacuation shelter and the tsunami arrival
(Unit) (Reference)		Degree [34]	Meter [15]	Meter Calculated ^b^	(1) Minute [29]	Minute [30]	Meter Calculated ^b^	(2) Minute [32]	(3) = (1) + (2) Minute Calculated	(4) Minute [15] [33]	(5) = (4)–(3) Minute Calculated
ID ^a^	3	6+	0.0–0.3	599	1.7	4.9	1,892	56.8	63.4	n/a	n/a
ID	8	6+	0.3–1.0	811	2.3	4.9	2,125	63.8	71.0	n/a	n/a
ID	14	7	0.3–1.0	951	2.6	4.9	2,096	62.9	70.5	n/a	n/a
ID	22	6+	0.3–1.0	2,694	7.5	4.9	4,543	136.4	148.8	60	−101.9
ID	27	7	0.3–1.0	1,324	3.7	4.9	1,418	42.6	51.2	60	7.8
ID	48	6+	0.3–1.0	1,748	4.9	4.9	1,336	40.1	49.9	60	10.1
ID	11	7	1.0–2.0	1,821	5.1	4.9	2,110	63.4	73.3	60	−13.3
ID	18	6+	1.0–2.0	795	2.2	4.9	265	8.0	15.1	50	34.9
ID	19	7	2.0–3.0	2,034	5.7	4.9	2,035	61.1	71.7	50	−21.7
ID	21	7	1.0–2.0	1,844	5.1	4.9	3,324	99.8	109.8	35	−87.3
ID	29	6+	2.0–3.0	840	2.3	4.9	1,682	50.5	57.7	n/a	n/a
ID	30	7	1.0–2.0	1,245	3.5	4.9	2,724	81.8	90.2	35	−67.7
ID	32	7	2.0–3.0	637	1.8	4.9	655	19.7	26.3	50	23.7
ID	39	6+	1.0–2.0	975	2.7	4.9	866	26.0	33.6	60	25.5
ID	46	6+	2.0–3.0	781	2.2	4.9	1,830	55.0	62.0	60	−4.3
ID	47	6−	2.0–3.0	1,118	3.1	4.9	483	14.5	22.5	60	37.5
ID	51	6+	1.0–2.0	1,146	3.2	4.9	3,909	117.4	125.5	35	−104.8
ID	5	6+	3.0–5.0	745	2.1	4.9	912	27.4	34.4	50	15.6
ID	34	6−	3.0–5.0	56	0.2	4.9	522	15.7	20.7	50	29.3
ID	49	6+	3.0–5.0	584	1.6	4.9	1,074	32.3	38.8	50	11.2
ID	50	6+	3.0–5.0	2,081	5.8	4.9	332	10.0	20.7	35	−8.2

^a^ Identification number of homecare recipients.

^b^ Calculated by QGIS.

n/a: Not applicable.

The homecare recipient who had the largest time difference was ID 51, whose residence was 1,146 m from the nearest visiting nursing station and 3,909 m from the welfare evacuation shelter. The time from departure of the nurse to arrival of the homecare recipient at the welfare evacuation shelter was estimated at 125.5 minutes. The visiting nurses supporting IDs 22 and 21 departed the station, which was more than 1,800 m from each of the two residences. The distance to the nearest welfare evacuation shelter was 4,543 m for ID 22 and 3,324 m for ID 21, and their evacuation times were more than 100 minutes. From a geographical point of view, it is possible for ID 19 to evacuate within a short time by traveling to a mountain behind the residence. However, considering the need for a power supply, ID 19 was required to evacuate to a welfare evacuation shelter. Additionally, the nearest welfare evacuation shelter was in an area with flood level 3, so that ID 19 needed to travel to a welfare evacuation shelter located about 2.5 km northeast. ID 19 took 71.4 minutes to evacuate and thus experienced a delayed escape. Other homecare recipients that would be delayed were IDs 11, 50, and 46, with IDs 11 and 46 both being more than 1,800 m from the nearest welfare evacuation center. Although ID 50 was only 332 m from the nearest shelter, the residence was close to the coastline and more than 2 km from the nearest visiting nursing station.

Meanwhile, ID 47 was estimated to arrive at a shelter with the most time to spare (37.5 minutes) before the arrival of the tsunami. Although ID 47 was fairly distant from the nearest nursing station, the residence was a short distance (483 m) from the shelter and away from the estuary. IDs 34, 39, 32, and 5 had the longest margins before the arrival of the tsunami and had a common locational characteristic for the distance from the nursing station to the residences, and their residences and welfare evacuation shelters were within 1,000 m of each other. Although IDs 48 and 27 were more than 1,000 m from the nursing station and shelter, they were close to the boundary line on the north side of the area of predicted tsunami inundation, estimated to arrive at the shelter within about 10 minutes.

### Sensitivity analysis

#### One-way sensitivity analysis

One-way sensitivity analysis revealed that the outcome was sensitive to the parameter “time that the 30-cm tsunami reaches the welfare evacuation shelter.” For half the base value of the tsunami arrival time, which is the lower limit of this parameter, 14 of the 21 homecare recipients (67.7%) had delayed evacuation, while four homecare recipients (19.0%) had delayed evacuation at twice the upper limit. Values changed by about 75 to 90 minutes for most homecare recipients.

The outcome was also sensitive to the parameter “travel speed from a residence to a shelter.” Twelve homecare recipients (57.1%) had delayed evacuation at 0.5 times the base value, while four homecare recipients (19.0%) had delayed evacuation at twice thus value. The maximum change was 204 minutes for ID 22.

The sensitivity of the outcome to the parameters “travel speed of the visiting nurse” and “time to move from a bed to wheelchair” was relatively low, and the number of delayed escapes was not affected by changing the value of either parameter. Changes were relatively small, being approximately 10 minutes (Table 2).

**Table 2:** One-way sensitivity analysis of the time difference between arrival at the evacuation shelter and tsunami arrival and the number of delayed escape scenarios (unit: minute).

Parameter		(1) Travel speed of a visiting nurse		(2) Time required to move from a bed to wheelchair		(3) Travel speed from a home of homecare recipient to a welfare evacuation shelter		(4) Time for a 30-cm tsunami to reach a welfare evacuation shelter	
	Range (times)	Slow 0.5	Fast 2.0	Long 2.0	Short 0.5	Slow 0.5	Fast 2.0	Short 0.5	Long 2.0
ID ^a^	3 ^b^	n/a	n/a	n/a	n/a	n/a	n/a	n/a	n/a
ID	8 ^b^	n/a	n/a	n/a	n/a	n/a	n/a	n/a	n/a
ID	14 ^b^	n/a	n/a	n/a	n/a	n/a	n/a	n/a	n/a
ID	22	−96	−85	−94	−86	−225	−21	−119	−29
ID	27	5	11	4	11	−34	30	−21	69
ID	48	5	13	5	13	−30	30	−20	70
ID	11	−18	−11	−18	−11	−77	18	−43	47
ID	18	33	36	30	37	27	39	10	85
ID	19	−27	−19	−27	−19	−83	9	−47	28
ID	21	−80	−72	−80	−72	−175	−25	−92	−40
ID	29 ^b^	n/a	n/a	n/a	n/a	n/a	n/a	n/a	n/a
ID	30	−59	−53	−60	−53	−137	−14	−73	−20
ID	32	22	25	19	26	4	33	−1	74
ID	39	24	28	21	29	0 ^c^	39	−4	86
ID	46	−4	−1	−7	0 ^c^	−57	25	−32	58
ID	47	34	39	33	40	23	45	7	97
ID	51	−94	−89	−95	−88	−208	−32	−108	−55
ID	5	14	17	11	18	−12	29	−9	66
ID	34	29	29	24	32	14	37	4	79
ID	49	10	12	6	14	−21	27	−14	61
ID	50	9	17	9	17	4	19	−3	49
Number of delayed escape scenarios	No.	7	7	7	7	12	4	14	4
	(%)	33.3	33.3	33.3	33.3	57.1	19.0	66.7	19.0

^a^ Identification number of homecare recipients.

^b^ Residents living outside areas reached by a 30-cm tsunami.

^c^ Zero values are counted as delayed escapes.

n/a: Not applicable

#### Multi-way sensitivity analysis

In the worst case (i.e., scenario A, a combination of a low travel speed of the nurse rescuer, long transfer time, low travel speed to a shelter, and short tsunami arrival time), all homecare recipients living in the tsunami arrival area were estimated to have a delayed escape. In the best case (i.e., scenario P, a combination of a high travel speed of the nurse rescuer, short transfer time, high travel speed to a shelter, and long tsunami arrival time), there were no delayed evacuations.

We next confirmed the results of scenarios A, C, E, G, I, K, M, and O. In these scenarios, the parameter “time for a 30-cm tsunami to reach the welfare evacuation shelter,” whose value is difficult to change by adopting a countermeasure, was fixed at “short.” Among the comparisons of the four combinations having different values for “travel speed of a visiting nurse” (i.e., A vs. I, C vs. K, E vs. M, and G vs. O), three escape delays changed in the comparison of G versus O. In the comparison of the four combinations with different values of “time required to move from a bed to a wheelchair” (i.e., A vs. E, C vs. G, I vs. M, and K vs. O), escape delays changed for one, two, one, and five homecare recipients, respectively. In the comparison of the four combinations with different values of “travel speed from the home of a homecare recipient to a welfare evacuation shelter” (i.e., A vs. C, E vs. G, I vs. K, and M vs. O), escape delays changed for five, six, five, and nine homecare recipients, respectively.

We then compared the results of the eight scenarios C, D, G, H, K, L, O, and P, for which the parameter of “travel speed from the home of a homecare recipient to a welfare evacuation shelter,” which is a sensitive parameter, was fixed at “fast.” There were 12, 10, 12, and 7 delayed escapes, respectively, in scenarios C, G, K, and O with a fixed value of “short” for “time for a 30-cm tsunami to reach a welfare evacuation shelter.” Among the four scenarios K, L, O, and P, for which the parameter of “travel speed of a visiting nurse” was fixed at “fast,” there were 12 and 7 delayed escapes in scenarios K and O, respectively (Table 3).

**Table 3:** Multi-way sensitivity analysis of the time difference between arrival at the evacuation shelter and tsunami arrival and the number of delayed escape scenarios (unit = minute).

Parameter		Range (times)															
(1) Travel speed of a visiting nurse		Slow 0.5								Fast 2.0							
(2) Time required to move from a bed to a wheelchair		Long 2.0				Short 0.5				Long 2.0				Short 0.5			
(3) Travel speed from the home of homecare recipient to a welfare evacuation shelter		Slow 0.5		Fast 2.0		Slow 0.5		Fast 2.0		Slow 0.5		Fast 2.0		Slow 0.5		Fast 2.0	
(4) Time for a 30-cm tsunami to reach a welfare evacuation shelter		Short 0.5	Long 2.0	Short 0.5	Long 2.0	Short 0.5	Long 2.0	Short 0.5	Long 2.0	Short 0.5	Long 2.0	Short 0.5	Long 2.0	Short 0.5	Long 2.0	Short 0.5	Long 2.0
Scenario pattern		A	B	C	D	E	F	G	H	I	J	K	L	M	N	O	P
ID^a^	3 ^b^	n/a	n/a	n/a	n/a	n/a	n/a	n/a	n/a	n/a	n/a	n/a	n/a	n/a	n/a	n/a	n/a
ID	8 ^b^	n/a	n/a	n/a	n/a	n/a	n/a	n/a	n/a	n/a	n/a	n/a	n/a	n/a	n/a	n/a	n/a
ID	14 ^b^	n/a	n/a	n/a	n/a	n/a	n/a	n/a	n/a	n/a	n/a	n/a	n/a	n/a	n/a	n/a	n/a
ID	22	−268	−178	−63	27	−260	−170	−56	34	−256	−166	−52	38	−249	−159	−44	46
ID	27	−72	18	−9	81	−65	25	−1	89	−67	23	−3	87	−59	31	4	94
ID	48	−70	20	−10	80	−62	28	−2	88	−62	28	−2	88	−55	35	5	95
ID	11	−117	−27	−22	68	−109	−19	−14	76	−109	−19	−14	76	−102	−12	−7	83
ID	18	−5	70	7	82	2	77	14	89	−2	73	10	85	6	81	17	92
ID	19	−118	−43	−27	48	−111	−36	−19	56	−110	−35	−18	57	−102	−27	−11	64
ID	21	−202	−150	−52	0	−195	−142	−45	7	−195	−142	−45	8	−187	−135	−37	15
ID	29	n/a	n/a	n/a	n/a	n/a	n/a	n/a	n/a	n/a	n/a	n/a	n/a	n/a	n/a	n/a	n/a
ID	30	−163	−110	−40	12	−155	−103	−33	20	−158	−105	−35	18	−150	−98	−28	25
ID	32	−28	47	2	77	−20	55	9	84	−25	50	4	79	−18	57	12	87
ID	39	−37	53	2	92	−30	60	9	99	−33	57	6	96	−26	64	13	103
ID	46	−94	−4	−12	78	−87	3	−4	86	−91	−1	−8	82	−83	7	−1	89
ID	47	−15	75	7	97	−8	82	14	104	−10	80	11	101	−3	87	19	109
ID	51	−233	−181	−57	−5	−226	−174	−50	2	−229	−176	−53	0	−221	−169	−45	7
ID	5	−44	31	−3	72	−36	39	5	80	−41	34	0	75	−33	42	8	83
ID	34	−16	59	7	82	−9	66	14	89	−16	59	7	82	−9	66	15	90
ID	49	−53	22	−4	71	−45	30	3	78	−50	25	−2	73	−43	32	6	81
ID	50	−24	29	−9	44	−16	36	−1	51	−15	37	0	52	−8	45	7	60
Number of delayed escape scenarios ^c^	No	17	7	12	2	16	6	10	0	17	7	12	1	16	6	7	0
	%	81.0	33.3	57.1	9.5	76.2	28.6	47.6	0.0	81.0	33.3	57.1	4.8	76.2	28.6	33.3	0.0

^a^ Identification number of homecare recipients.

^b^ Residents living outside areas reached by a 30-cm tsunami.

^c^ Zero values are counted as delayed escapes.

n/a: Not applicable

## Discussion

### Validity of simulation

We confirmed that the actual positions did not deviate after converting addresses to geographical data and plotting them on a GIS map. We calculated the shortest distance using the GIS for the routes traveled by visiting nurses and the routes to welfare evacuation shelters and then visually confirmed the validity of the route. Therefore, the routes and distances used to calculate the travel times are reasonable and accurate.

Sensitivity analysis showed that differences in speed due to an increase or decrease in traffic volume do not significantly affect the main outcome. However, there will be congestion if many people are evacuating by private car during a disaster, and there is thus a possibility that vehicles will move at a much lower speed than that assumed in this research.

Approximately 90% of homecare recipients that we surveyed to determine parameters were bedridden, with about 17% using ventilators (which are criteria used in determining the daily-life independence level [35]). In the present study, 85.7% were bedridden, which is comparable to the percentage in the previous study. Evacuation requires tasks such as attaching equipment, including ventilators and suction equipment installed on the bedside, to the wheelchair. It is therefore estimated that the time required for transfer assumed in this study will be slightly longer than that determined in the previous study. In particular, this could affect the outcome when the difference between the time required for evacuation and the arrival time of the tsunami is within several minutes.

Because the homecare recipients in the present study will also carry medical equipment, the travel speed in the situations assumed in this study will be lower than that indicated in previous studies. The present study did not consider whether it was actually possible to push a wheelchair carrying a homecare recipient and a medical device. It is therefore necessary to improve the validity of this aspect of the simulation in future work.

We used values estimated by the Japanese government as the predicted times for a tsunami having a flow depth of 30 cm to reach each welfare evacuation shelter. The values are estimated according to the speed of the tsunami generated by the seismic intensity of the very large East Japan Great Earthquake (magnitude 9) and the latest topographical data, and they are reasonably used in the present study.

### Factors contributing to delayed escape

In this research, we assumed that residents will cooperate and allow themselves to be evacuated in accordance with the policy of the Japanese government. Thus, other factors were found to cause escape delay, specifically to live near the coast and to live far from the evacuation shelter. Of course, the positional relationship between coast, residents, and evacuation centers varies from place to place, but it is common that their positional relationship strongly influences the success rate of evacuation. Moreover, their positional relationship can be confirmed by GIS. Details are as follows.

Almost all of homecare recipients who had delayed evacuation (i.e., IDs 11, 19, 21, 22, 30, 46, 50, and 51) were close to the coast or needed to move at least 1.8 km to a shelter. All homecare recipients in the Great East Japan Earthquake of 2011, elderly people who had difficulty in reaching high ground, evacuated to shelters on the plains and were killed [36]. According to a survey on evacuation behaviors in the city of Ishinomaki, about 15% of deaths were due to victims being bedridden, about 4% were due to victims waiting to be picked up, and about 21% were due to events during the process of evacuation [37]. In other words, the distances from the homecare recipient’s residence to the rescuer and the welfare evacuation shelter and the travel speed were directly related to the time of arrival at the welfare evacuation shelter and can be considered important factors contributing to the escape delay.

IDs 11, 27, 39, 48, 21, 30, and 51 needed to travel more than 1 km toward the estuary. As a tsunami countermeasure, the Japanese government has instructed people to immediately leave coastal areas to reduce the chance of human injury when they feel a strong earthquake [38]. However, because rescuers of these homecare recipients are close to estuaries, the evacuation will involve movement toward the coast. In other words, the location of welfare evacuation shelters could trigger evacuation behaviors that increase proximity to the tsunami inflow and increase the risk of escape delays.

### Recommendations for disaster countermeasures

For homecare recipients, evacuation shelters that secure a power supply and continue medical procedures are indispensable. Also, living far from the shelter will increase the risk of escape delay. Therefore, welfare evacuation shelters in a region should be designated based on the proximity of homecare recipients. Sensitivity analysis showed that the outcome had a high sensitivity to the travel speed. Accelerated movement is expected to reduce the number of escape delays. However, there is a limit to the speed of moving with a wheelchair while operating the medical equipment during a disaster. Therefore, to avoid escape delays, measures are needed to shorten the distances from rescuers to homecare recipients and welfare evacuation shelters. In other words, a municipality should identify the locations of residences of homecare recipients in their area and designate welfare evacuation shelters considering the inundation time of the tsunami, distance from the rescuer to the homecare recipient, and the time required for a rescuer to take the homecare recipient to the shelter. In addition, it is necessary to identify homecare recipients who live in areas from where it is difficult to evacuate before the tsunami arrives and to take countermeasures. As a means to make it easiest to implement, we recommend relocating homecare recipients as close to evacuation shelters as possible.

Another way to reduce escape delay is to shorten the time needed for the rescuer to reach the homecare recipient. If a neighbor performs the rescue, they will arrive at the recipient’s home quicker and shorten the evacuation time. However, since it is necessary for a neighbor to operate the medical equipment, prior training is needed with the cooperation of doctors and nurses.

Periodic evacuation drills are needed to support homecare recipients in evacuating effectively. Individual training according to the individual physical condition is necessary, in parallel with uniform training in which the general population moves toward designated evacuation shelters in unison. Sullivan et al. stated that it is necessary to improve judgment skills through evacuation training for disabled people to evacuate without confusion [39]. Because in our scenario a homecare recipient is expected to move to an evacuation shelter after a visiting nurse arrives, the homecare recipient and visiting nurse need to train to determine the evacuation method by sharing the best route for evacuation. Also, training needs to be done in consideration of the time of day in which the disaster occurs. It is necessary to prepare equipment, such as lights to be used in the event of a power outage at night. Considering differences in the seasonal environment and supplementary warming countermeasures for people sensitive to low temperatures, we think that it is preferable to train during every season. Training is effective for improving the speed of travel to the welfare evacuation shelter and helping the rescuer make quicker decisions according to the situation; for example, if it can be predicted that the flow depth of the tsunami is shallow and the risk of the homecare recipient collapsing at home is low, it will be possible to make a decision to stay at home and not evacuate.

Finally, helicopters could provide a means of transport during a disaster. Problems with helicopter transportation by ventilator users are as follows: disaster prevention helicopters are not designed to move artificial ventilation users, helicopters lack a stable power supply, the transport tremors and vibrations are large, and the increased altitude decreases oxygen partial pressure [19]. However, a helicopter can reach a patient quickly even after infrastructure is destroyed. We think that we should address the helicopter transport drawbacks and consider it as a possible means of disaster escape.

### Usefulness of a GIS

The simulation of evacuation behavior using a GIS is effective for decision making when planning for evacuation. Previous studies have demonstrated the effectiveness of a GIS in terms of evacuation behaviors and infrastructure development [40], such as the determination of the necessity of evacuation and selection of an evacuation site in a coastal area [41]. In the present study, we showed that a GIS is effective for determining evacuation behavior. If flooding is deep in the middle of an evacuation route, for example, a decision can be made whether to evacuate given the flooding’s impact on mobility. In this way, by concatenating multiple pieces of information on a map using a GIS, a concrete evacuation behavior simulation is realized, and the simulation is considered to be effective in terms of the decision making of a homecare recipient.

In this research, we used free GIS software with open-source data. If someone can obtain the address of the people who need rescue, they can reproduce our method for locations anywhere in the world.

The Global Positioning System (GPS) is used for disaster countermeasures [42]. In Japan, since the Great East Japan Earthquake, development of a system linking GIS and GPS has been underway [43]. In this research, we used GIS as “preparation” for disaster; however, if we could link GPS and GIS, we could instantaneously grasp the position of rescuers and homecare recipients in case of disaster, and increase the speed of rescue. For example, the visiting nurse nearest to the home care recipient could be designated as the rescuer, thereby shortening the evacuation time.

For homecare recipients who have difficulty in leaving home, the method is expected to be applied to virtual evacuation drills that combine information obtained by a GIS and virtual reality (VR). In recent years, a virtual tsunami evacuation training system using smart glasses for disaster prevention education has been developed [44]. By combining virtual tsunami inundation information with images of real evacuation routes determined by a GIS, it would be possible for homecare recipients who have difficulty in leaving home to take part in virtual evacuation drills. Specific methods are as follows: (1) Calculate the appropriate evacuation route and reproduce the scenery on the route with VR. (2) By evacuating by VR, verify the usability of the evacuation route. (3) Have the evacuation done without showing the evacuation route with VR. (4) After completing the evacuation, show the participants whether they passed the correct escape route by overlaying the evacuation route and the calculated optimal evacuation route. (5) Test evacuation response according to various situations by setting unusable routes and changing evacuation means. The advantage of this method is that it makes it possible to practice evacuations individually, rather than mass evacuation drills. Inclusion of people who cannot participate in actual evacuation drills due to disease or disability is also possible.

### Limitations of the present study and directions of future study

The one-way sensitivity analysis revealed that the parameter with the highest impact on the outcome was the tsunami arrival time. It is difficult to accurately predict the arrival time of a tsunami, but it is a core part of disaster simulation. To improve the validity of simulation, it is desirable to predict the arrival time of a tsunami with high accuracy.

Traffic congestion during a disaster was not considered in the time calculated for the visiting nurse to travel to the homecare recipient’s home. To improve the validity of this travel time, it is necessary to simulate traffic congestion during a disaster. To improve the adequacy of the time available to transfer a homecare recipient from the bed to a wheelchair, it is necessary to perform demonstration experiments for transfers of homecare recipients with medical equipment, such as a ventilator. In addition, a similar experiment is needed for the case where a nurse pushes a homecare recipient in a wheelchair equipped with a medical device, to obtain the actual travel speed. Moreover, a demonstration experiment is needed to obtain the distance that the evacuee and rescuer are capable of traveling when the rescuer pushes the evacuee in a wheelchair.

## Conclusions

1. There are vulnerable groups in society that must be provided additional assistance during disaster evacuations.
2. One group most affected comprises individuals in need of medical treatment using externally powered electric devices and living far from a welfare evacuation shelter.
3. This group needs support and education.
4. The outcome of the disaster is influenced by the speed of its occurrence, the status of homecare recipients, the number of available staff, and the time required to rescue them. In particular, the outcome is affected by the distance between the rescuer and the evacuee and shelter.
5. We can predict the time window during which we can respond to a disaster, identify the affected people through risk and vulnerability analysis, schedule staff so that enough rescuers are available, and, finally, analyze the routes and types of transportation.
6. What GIS can give us is the localization of the people and, based on the geographical data, areas that can be affected most by a tsunami.
7. The simulation of evacuation behavior using a GIS is effective for decision making when planning for evacuation. By linking GIS with GPS, there is a possibility of increasing the speed of rescue activities.
8. The most immediately viable means is relocating homecare recipients as close to safe welfare evacuation shelter as possible. There is a possibility of transport by helicopter.
